# A Review of the Current Status of Peste des Petits Ruminants Epidemiology in Small Ruminants in Tanzania

**DOI:** 10.3389/fvets.2020.592662

**Published:** 2020-11-25

**Authors:** Enokela S. Idoga, Bryony Armson, Ruth Alafiatayo, Adah Ogwuche, Erik Mijten, Abel B. Ekiri, Gabriel Varga, Alasdair J. C. Cook

**Affiliations:** ^1^Department of Veterinary Physiology, Biochemistry and Pharmacology, University of Jos, Jos, Nigeria; ^2^vHive, School of Veterinary Medicine, University of Surrey, Guildford, United Kingdom; ^3^Zoetis-ALPHA Initiative, Zoetis, Zaventem, Belgium

**Keywords:** peste des petit ruminants, PPRV, small ruminant morbillivirus, sheep, goats, small ruminant

## Abstract

Peste des petits ruminants (PPR) is a highly contagious viral disease of sheep and goats with high mortality. The disease is of considerable economic importance in countries such as Tanzania, where small ruminant products are important for sustainable livelihoods. This review assesses current knowledge regarding the epidemiology of PPRV in Tanzania, highlighting the challenges with respect to control and suggesting possible interventions. Thirty-three articles were identified after literature searches using Google Scholar and PubMed. Studies revealed that PPRV is endemic in sheep and goats in Tanzania, although seropositivity has also been reported in cattle, camels, buffalo, Grant's gazelle, wildebeest and impala, but with no clinical manifestation. Three lineages (lineage II to IV) of PPRV have been identified in Tanzania, implying at least two separate introductions of the virus. Diagnosis of PPR in Tanzania is mostly by observation of clinical signs and lesions at post mortem. Risk factors in Tanzania include age, sex, species, and close contact of animals from different farms/localities. Although there is an efficacious vaccine available for PPR, poor disease surveillance, low vaccine coverage, and uncontrolled animal movements have been the bane of control efforts for PPR in Tanzania. There is need for collaborative efforts to develop interventions to control and eradicate the disease. The establishment of a national reference laboratory for PPR, conduct of surveillance, the development of high-quality DIVA vaccines, as well as execution of a carefully planned national vaccination campaign may be key to the control and subsequent eradication of PPR in Tanzania and achieving the global goal of eradicating PPR by 2030.

## Introduction

Peste des petits ruminants (PPR) is a highly contagious and acute viral disease of sheep and goats, with sub-clinical manifestation in cattle, pigs, and camel. The disease has also been reported in some wildlife species including Dorcas gazelles (*Gazella dorcas*) (1), Nubian ibex (*Capra nubiana*), Laristan sheep (*Ovis vignei laristanica*), and gemsbok (*Oryx gazelle*) (2). The disease is characterized by fever, anorexia, nasal and ocular discharges, sores in the mouth, pneumonia, profuse diarrhea, and often death (3). Reported morbidity and mortality rates have varied between 90–100% and 50–100%, respectively (2). PPR has also been associated with a high rate of abortion in infected goats (4). Consequently, PPR is a major constraint to small ruminant production in Africa (5, 6) and is thus of high economic importance, especially in areas with a high reliance on small ruminant products (7).

PPR is caused by peste des petits ruminants virus (PPRV), species *Small ruminant morbillivirus* (SRMV), a member of the genus *Morbillivirus*, in the family *Paramyxoviridae* (8, 9). It is closely related to other members of the genus, including rinderpest virus, measles virus, and canine distemper virus (8, 10). The virus is highly contagious, easily transmitted by direct contact of healthy animals with the secretions and/or excretions from infected animals, or by contact with infected fomites (2, 11). PPRV exists as one serotype, but sequence analysis of the nucleoprotein (N) gene and the fusion protein (F) gene has revealed four genetically distinct lineages (10, 12). Lineages I and II are mainly found in West and Central Africa; lineage III is found mainly in East Africa, Yemen and Oman; and lineage IV is found across the Arabian Peninsula, the Middle East, southern Asia and recently, in several African territories (10, 13, 14).

The geographical spread of PPR is wide. The disease was first identified in West Africa in the 1940s (15, 16), and has since been observed in North and Central Africa, the Middle East, and parts of East Africa and Asia (17, 18) and Europe (19). In East Africa, PPRV was first isolated in Ethiopia in 1991 (20), although sick goat herds in the Afar region of Ethiopia were suspected to have PPR much earlier in 1977 (21, 22). In Tanzania, PPR was officially confirmed in 2008 (23, 24). However, Karimuribo et al. (23) suggested that the disease had been in circulation in Tanzania for at least 4 years previously, as farmers had reported “rinderpest-like” syndromes in domestic small ruminants, supported by clinicopathological reports and sero-prevalence data. PPR has since been reported in goats, sheep, and camels in Tanzania (25–28).

Similar to other African countries, the impact of PPR on agriculture in Tanzania has wide implications. Agriculture is a mainstay of Tanzania's economy, with approximately one fifth of the agriculture-derived economy emanating from the livestock subsector (29, 30). About 22% of total household income in Tanzania is from livestock rearing, and ~60% of rural household incomes come from livestock activities (29). Cattle, goats, and sheep constitute a large share of the animals reared by Tanzanian households as sources of protein and livelihood (31), with sheep and goats accounting for about 22 percent of meat consumed in Tanzania (32). Goat and sheep are the species of choice for pastoralists, due to their hardiness and ability to withstand the harsh arid and semi-arid climates. They are mostly kept under extensive management systems with communal grazing and sometimes housing (32).

Currently a global initiative driven by the Food and Agriculture Organization of the United Nations (FAO) and the World Organisation for Animal Health (OIE) exists to eradicate PPR by 2030 (33). For this to be attainable, it is important to understand the specific epidemiological features of the disease and identify the socio-economic factors that must be considered to stop the transmission of the disease (34). This review is aimed at updating knowledge on the epidemiology of PPR in Tanzania, one of the focus countries for the African Livestock Productivity and Health Advancement (A.L.P.H.A.) initiative, which aims to advance livestock health and productivity in sub Saharan Africa. This article investigates the occurrence and distribution of PPR in Tanzania, the circulating strains, risk factors, economic impacts, control and prevention strategies, and challenges to control of PPR. Additionally, this review aims to identify the challenges and research gaps to inform future control efforts, so that small ruminant production may be improved in this region of East Africa.

## Methods

Literature searches were conducted in PubMed and Google Scholar. Grey literature was obtained using Google Search and the official websites of FAO and OIE (www.fao.org and www.oie.int). The search terms used were “PPR Tanzania” and “Peste des petits ruminants AND Tanzania.” All searches were carried out between September 2019 and July 2020. First, title and abstract were reviewed to determine their eligibility. For eligible articles, full text was subsequently reviewed while non-eligible articles were excluded.

**Figure 1:**
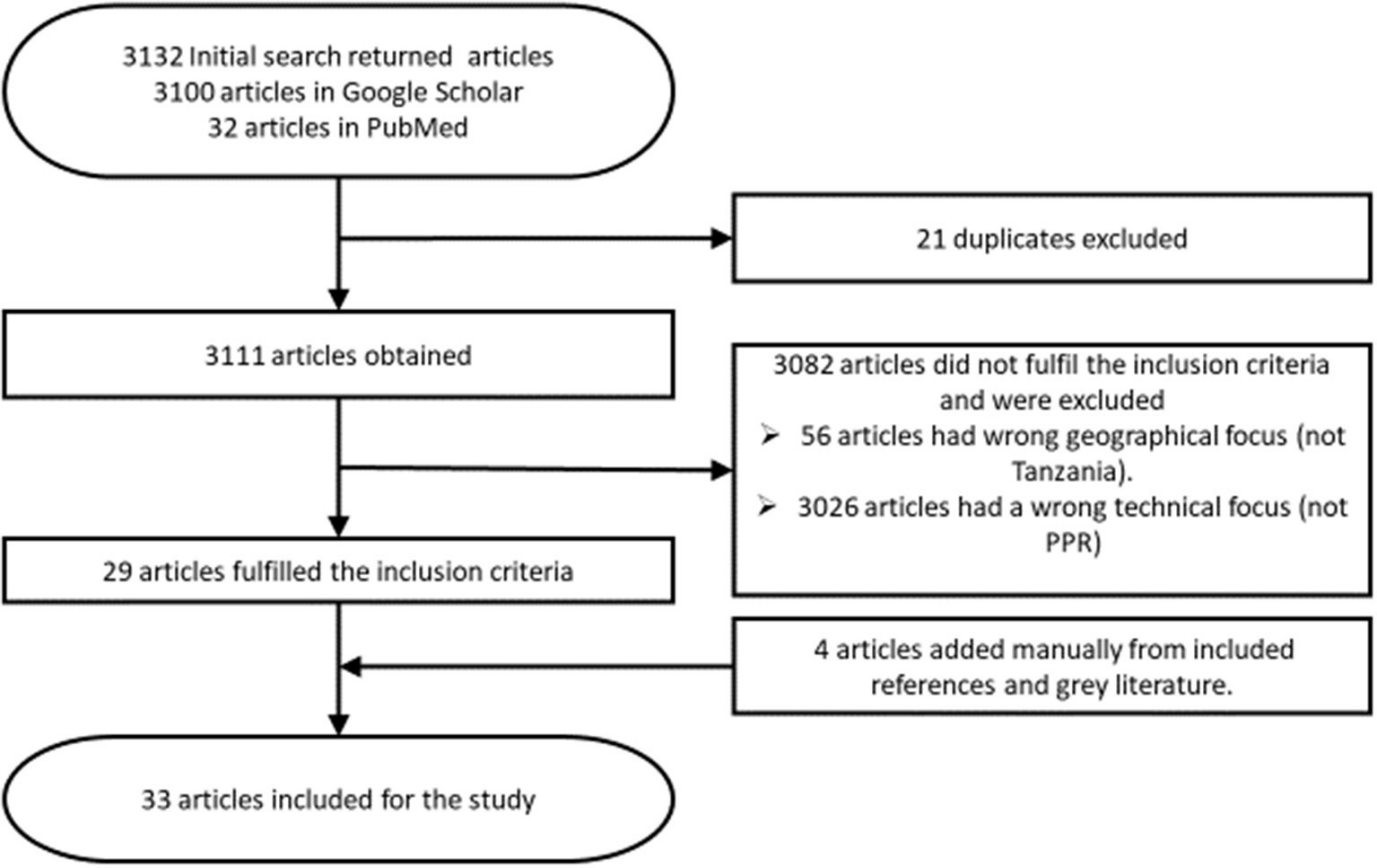
Flow diagram of the systematic review and identification of eligible articles.

Eligible articles were those published about peste des petits ruminants in Tanzania within the last 16 years (2004–2020), published in or translated to the English language. Only articles concerning case reports, reviews, outbreaks, risk factors, economic losses, control measures, and prevalence of PPR in Tanzania were considered relevant. Additionally, conference papers and theses relating to the topic were included if they were not published in a peer reviewed journal at the time of review. Articles were excluded if they had a geographical focus other than Tanzania or focused on a different disease. Editorials, letters to the editor, opinions or commentaries without original data were also excluded. Data extracted from eligible articles included clinical signs, diagnosis, occurrence, distribution and circulating strains, risk factors, economic losses, control, prevention, and challenges of PPR in Tanzania. The process through which articles were sourced, identified, and selected for this review is shown in Figure 1.

## Results

### Selected Studies

Thirty-three articles were eligible for this review, 24 were research articles, and one was a review article (Supplementary Material 1). Additionally, there were two conference papers, four theses, and two technical reports.

### Clinical signs

Two studies described the clinical signs of PPR in Tanzania and suggest that goats were more susceptible to PPR than sheep, with sheep exhibiting a milder form of the disease (14, 35). The main symptoms of PPR described included anorexia, emaciation, severe depression, fever (40–41°C), diarrhea, muco-purulent nasal, and ocular discharge and erosive and necrotic stomatitis (14, 35). Abortion and nodular lesions were also observed, which were not reported to be common in neighboring Kenya (35). Additionally, when performing post-mortem examination of confirmed cases of PPR, Muse et al. (14) observed lung congestion and consolidation, and increased thickness of inter-alveolar walls, indicating pneumonia.

### Diagnosis

In the reviewed studies, diagnosis of PPR in Tanzania was mostly by observation of clinical signs and lesions at post-mortem, followed by monoclonal antibody-based competitive enzyme-linked immunosorbent assay (cELISA) for the detection of PPRV antibodies to determine a previous or current infection (26, 36–40, 50). Additionally, some of the studies also utilized confirmatory molecular methods for the detection of PPRV genome (27, 36, 41–43).

Samples collected for testing included swabs of conjunctival, nasal and oral discharges and ulcers, whole blood, and serum samples for serology (27, 36, 41–43). Portions of intestines, lungs, and lymph nodes were also collected and homogenized for the detection of viral RNA (41, 42). Real-time reverse transcription polymerase chain reaction (rRT-PCR), targeting the PPRV nucleoprotein (N) gene, was used to identify the presence of PPRV genome in buffy coat, homogenized tissue samples, and nasopharyngeal and ocular swabs of suspected cases (12, 27, 36, 41–45). Additionally, phylogenetic analysis based on the N gene has been utilized to determine the PPRV lineage and to establish epidemiological relationships (12, 36, 41, 44). The immunocapture enzyme-linked immunosorbent assay (IC-ELISA) for the rapid identification of PPRV antigen (46), recommended by OIE (47), was not reported to have been used in any of the reviewed articles.

Serological tests performed in the reviewed studies were mostly ELISA techniques such as the competitive PPRV specific anti-H monoclonal based ELISA (c-ELISA) as recommended by the OIE (27, 28, 39, 43, 48–50). The c-ELISA detects antibodies to confirm that the animal has been exposed to PPRV at some point in their lifetime. However, due to the vaccines currently used in Tanzania (live attenuated Nigerian strain 75/1 vaccine) these tests are not able to differentiate between previously infected or vaccinated animals (51).

### Occurrence and Distribution

Seven studies reported the occurrence and distribution of PPR in Tanzania (12, 24–26, 43, 49, 52). The studies show PPR to be endemic in goat and sheep populations throughout Tanzania, with several outbreaks reported in different regions of the country (26, 43). Limited evidence of PPRV infection has been observed in wild small ruminants (such as dik-dik, gazelle etc.) and these were reported to be restricted to areas in close proximity with livestock in the Serengeti ecosystem of northern Tanzania, indicating a spill over of infection from livestock populations in Ngorongoro district (24, 26, 52). Seropositivity without clinical manifestation has been observed in cattle, camels, buffalo, Grant's gazelle (*Nanger granti*), wildebeest, and impala sampled in Ngorongoro district in northern Tanzania (24, 25, 52).

### Outbreak History

Eight of the selected studies discussed events that surround the history of PPR outbreaks in Tanzania. Following the serological evidence of PPRV infection in Kenya and Uganda in 1994, the first nationwide serological screening was performed in Tanzania in 2000. Over 3,000 serum samples were screened for PPRV antibodies using the competitive ELISA (cELISA) and all cELISA results were negative (26, 41). A confirmed PPR outbreak in Kenya in August 2006, coupled with reports of clinical signs resembling PPR and high mortality amongst sheep and goats in Ngorongoro, northern Tanzania in December 2007 prompted another investigation (36, 49). Clinical and pathological investigations performed in the Ngorongoro district in March 2008 yielded inconclusive results from 112 sheep and goats, whilst serological investigation was negative for PPR (36). As high mortality persisted amongst the sheep and goat populations in Ngorongoro and the neighboring Mara district, a new investigation confirmed the presence of PPR in Ngorongoro in June 2008, where 129/404 serum samples tested positive for PPR antibody (26, 36). Phylogenetic analysis of isolated PPRV from this investigation identified it as a member of lineage III, the most abundant lineage in eastern Africa (36). Spiegel and Havas (53) suggested that the emergence of PPR in Tanzania in 2008 may have been related to the humanitarian crisis in Kenya in 2007, caused by a highly contested election that led to widespread violence and the displacement of citizens into refugee camps in northern Tanzania. This may have contributed to the introduction of PPRV to Tanzania, due to increased transboundary animal and human movement (2). However, retrospective serological analysis performed by Karimuribo et al. (23) using serum samples collected in 2004 suggested the presence of PPRV in northern Tanzania before 2008, and therefore the time of the true emergence of PPR in Tanzania is unknown.

It was believed that PPR was confined to northern Tanzania until 2009 (42). Negative results were observed in retrospective serological analysis performed using archived sera samples collected from small ruminants for Rift Valley fever surveillance in Mtwara and Lindi regions of southern Tanzania in 2007. Although the sampling strategy of this study was not adequate to confirm absence of infection, these results support the theory that PPR may have been introduced in these regions thereafter (54). PPR was first reported in southern Tanzania in December 2009, in Likuna, a village in the southern Newala district, suspected to be transmitted via goats purchased for Christmas and New Year festivities from Pugu livestock market in the outskirts of Dar es Salaam (14, 36). Since then, outbreaks of PPR have been reported in Tandahimba and Newala districts of Mtwara region of southern Tanzania in 2011 (43), in Ngorongoro and Mvomero districts in northern and eastern Tanzania (respectively) in 2012 (41), and in the Loliondo area in Ngorongoro district of Northern Tanzania in 2016 (27).

### Sero-Prevalence

The sero-prevalence of PPR in Tanzania was reported in six of the studies performed between 2008 and 2016 and results are summarized in Table 1. The national prevalence of PPR was estimated in a study performed using samples collected in 2013 and 2015 as 26.0% with a true prevalence estimated as 27.1% (95% confidence interval: 25.6–28.5%), although prevalence differed widely by region, varying from 2.6% in Katavi region to 67.3% in Arusha and 70.0% in Morogoro (49). Indeed, the authors suggested that the high sero-prevalence observed may have been due to previous PPR vaccination in these regions. A study performed by the same authors in 2016 (27) also observed a high sero-prevalance (74.6%) in Arusha region, however, they reported no history of PPR vaccination, according to records from the District Veterinary Office.

**Table 1:** Sero-prevalence of PPR reported in Tanzania.

**Article**	**Location**	**Region/district**	**Study period**	**Overall prevalence (p/n)**	**Prevalence in goats (p/n)**	**Prevalence in sheep (p/n)**
Swai et al. (48)	Northern Tanzania	Ngorongoro, Monduli, Longido, Karatu, Mbulu, Siha, and Simanjiro districts	2008	45.8% (704/1,549)	49.5% (443/892)	39.8% (262/657)
Muse et al. (43)	Southern Tanzania	Tandahimba and Newala districts of Mtwara region	2011	31.0% (67/216)	35.3%^a^	30.7%*
Kgotlele et al. (49)	Across Tanzania	118 villages in 14 regions across Tanzania	2013, 2015	26.0% (998/3,838)	26.3% (759/2,886)	25.2% (240/952)
Torsson et al. (50)	Northern Tanzania	Ngorongoro district Ulanga district, Kilombero district, and Mvomero district	2014, 2015	46.8% (223/476) (2014), 10.0% (48/481) (2015)	48.3% (115/238) (2014), 10.8% (35/323) (2015)	45.5% (108/238) (2014), 8.2% (13/158) (2015)
Herzog et al. (39)	Northern Tanzania	Arusha and Manyara Regions	2016	21.1% (1580/7,496) (including cattle) 27.6% (1241/4,499) (for goats and sheep only)	28.8% (696/2,419)	26.2% (545/2,080)
Kgotlele et al. (27)	Northern Tanzania	Loliondo area in Ngorongoro district	2016	74.6% (179/240)	75.7% (137/181)	71.2% (42/59)
Mbyuzi et al. (54)	Southern Tanzania	Mtwara and Lindi regions, Tandahimba and Newala districts	2007, 2009	0% (2007), 27.8% (150/504) (2009)	0% (2007), 28.7% (125/434) (2009)	0% (2007), 35.7% (25/70) (2009)
Nkangaga et al. (28)	Western Tanzania	Kasulu, Kibondo and Kigoma in Kigoma region	2011–2012	5.1% (23/450)	4.8% (20/415)	8.6% (3/35)

a *Figures were not available for goats and sheep for (43)*.

*n, number of animals tested for PPRV antibodies*.

*p, number of animals that were positive for PPRV antibodies*.

Torsson et al. (50) observed a decrease in the sero-prevalence of PPR from 49.3% in 2014 to 10.0% in 2015, in a study performed at the wildlife–livestock interface in Ngorongoro district in the northern Arusha region, and Ulanga, Kilombero, and Mvomero districts in the south-eastern Morogoro region. The authors attributed the difference in sero-prevalence to vaccination that was performed in the Morogoro and Mtwara regions prior to sample collection in 2014, and therefore it is likely that the high seropositivity was influenced by vaccine-induced antibodies, compared with a population containing more naïve susceptible animals (3–12 months of age) during the 2015 sample collection.

Kgotlele et al. (49) reported that the sero-prevalence of PPR did not differ significantly between goat (26.3%) and sheep (25.2%) populations. However, Swai et al. (48) and Nkangaga et al. (28) observed a significantly higher sero-prevalence in goats when compared to sheep (Table 1).

### Circulating Strains of PPRV

Only 4/33 of the eligible studies characterized the strains of PPRV present in Tanzania. Kgotlele et al. (41) carried out phylogenetic analysis based on the N gene of PPRV, on nasal and ocular swabs and whole blood samples obtained from PPR cases in northern and eastern Tanzania. They identified lineage III, with a high genetic identity to PPRVs from Sudan and Ethiopia. Jones et al. (45) also identified PPRV lineage III in samples collected in Ngorongoro District in 2015, which clustered with isolates from Uganda, Kenya and Democratic Republic of Congo. Additionally, Misinzo et al. (12) identified lineage II and IV from goats in the 2011 PPR outbreak in southern Tanzania (52). Therefore, this suggests at least three separate introductions of PPR into Tanzania.

### Risk Factors

The risk factors for PPRV infection were investigated by eight of the eligible studies, using questionnaires and sero-prevalence data. The risk factors identified as major contributors to PPR occurrence in Tanzania included communal grazing and housing (14, 42, 55, 56); the practice of selling sick animals at cheap prices and bought by livestock keepers for slaughtering in other villages (14); the mixing of infected with healthy animals in markets; and poor access to veterinary services (14).

Torsson et al. (50) reported that female sheep and goats may be at higher risk of PPR than males because they are kept longer on the farms and therefore have a longer risk period for PPRV exposure. Additionally, a higher prevalence of PPR was reported in pastoral (primarily livestock) management systems, compared to agropastoral systems (a mix of crop and livestock) in Northern Tanzania potentially indicating pastoral management as a risk factor (36, 39, 40, 48). Mbyuzi et al. (57) observed a significantly higher incidence of PPR as reported by farmers in the rainy than the dry season. Additionally, Mdetele et al. (58) reported a significantly higher seroprevalence of PPR in semi-arid and coastal agro-ecological zones in Tanzania, when compared to the plateau ecological zones, suggesting coastal, and semi-arid regions are high risk ecological zones. The practice of grazing sheep and goats in close proximity to or on wildlife grazing areas was also shown to increase the risk of PPR occurrence in wild ruminants (24, 52).

### Control and Prevention

PPR control programs initiated by the Tanzanian government were discussed by five of the reviewed studies. Between 2006 and 2008, an estimated 64,661 animals were culled in Tanzania, in attempts to control PPR (59). In response to the incursion of PPR in Tanzania in 2008, the United Republic of Tanzania Ministry of Livestock and Fisheries carried out mass (blanket) vaccination of sheep and goats in the Northern and Lake Zones bordering Kenya through the Vaccination for Control of Neglected Animal Diseases in Africa (VACNADA) project, funded by the European Union Food Facility (37). The VACNADA project achieved 71.1% seroconversion following vaccination, which according to Baron et al. (60), may have been enough to successfully prevent PPR transmission. Despite this, PPR was observed a few months later in southern Tanzania in 2009 and proceeded to spread across the country, including to northern Tanzania (14, 36, 42, 43). Since then several vaccination campaigns have been executed, including in northern Tanzania in 2010 (23), in small ruminants along livestock marketing routes in 2011, and in herds in the area around Mikumi National Park in 2013 (61). The Nigerian strain 75/1 PPR vaccine is often used for PPR control in Tanzania, and other Southern African Development Community (SADC) member countries (26, 41). Karimuribo et al. (23) reported that farmers in Tanzania used antibiotics to treat clinical cases of PPR in their flock.

### Challenges for the Control of PPR

Despite numerous vaccination campaigns, PPR has spread throughout most of Tanzania. Two articles outlined the challenges hindering the control of PPR in Tanzania. Torsson et al. (26) highlighted low awareness among small ruminant farmers, traders, and transporters; uncontrolled livestock movements; poor availability of diagnostic tools, poor surveillance and reporting; and a lack of capacity to enforce regulations as major constraints in the control of PPR. In addition to uncontrolled livestock movement, Kivaria et al. (36) reported that poor zoo-sanitary habits by farmers and a lack of proper local and national control strategies are the main factors responsible for the persistence of PPRV in Tanzania.

### Economic Impact

The economic losses attributed to PPR in Tanzania were reported by one grey literature report, two theses and a review article. Economic losses may be due to depletion of the small ruminant population, by mortalities associated with the disease, or by culling as a control measure (59). Other economic losses may result from the cost of medication, vaccination, veterinary and labor services, a reduced market value due to poor body condition, and the embargo on livestock markets imposed by authorities (44, 51, 59). A study in 2012 in Tandahimba and Ulanga districts in southern Tanzania found that the outbreaks of PPR reduced the average value of small ruminants by 10%, caused a decrease in flock size, and increased the inputs and risks of small ruminant production (26). This resulted in a loss of potential income and a reduced ability of the flock to support household livelihood (by ~30%). Consequently, the estimated total loss of income to PPR was estimated to be TZS 335,420 (155 Euro) per household per year, amounting to a cumulative national loss in excess of TZS 200 billion (92 million Euro) per year (26).

## Discussion

There is a dearth of literature on the status of PPR in Tanzania, indicated by the low number of eligible articles obtained for this review. Reviewed studies have shown that the incursion of PPR into Tanzania in 2008 may be directly linked with the emergence and spread of PPR in neighboring Kenya in 2006 (53). A pointer to this is the fact that the first report of PPR in Tanzania was an outbreak in Ngorongoro district, bordering Kenya (36, 53), and the strain of PPRV isolated belonged to lineage III, the same lineage predominant in Kenya, and other countries in East Africa at that time (36, 62). Subsequent isolation of PPRV belonging to lineage II and IV (12, 52) suggest that PPRV may have been imported into the country on more than one occasion (12, 36). Lineage II PPRV in Tanzania may have come from Uganda (12, 36), however, the origin of Lineage IV may be difficult to discern as it is widely spread across the world and in East Africa (63). Of the eight countries bordering Tanzania, PPR has been reported in four: Kenya, Uganda, Democratic Republic of Congo and Burundi (64, 65). Indeed, the existence of an informal cross border livestock trade in the eastern and southern African regions (66, 67) presents a continuous risk of PPR incursion, persistence and spread among these countries and beyond (51, 65, 68, 69).

Studies reviewed show that PPR is endemic throughout Tanzania, and it has had devastating effects on the small ruminant population and the livelihoods of pastoralists across the country over the last several years (36). This is attributable to the high transmissibility and morbidity of PPR (2), which has resulted in its rapid spread in small ruminant populations through large areas of Africa and Asia within the past 20 years (70). Evidence of interspecies transmission of PPR has been observed in several studies (1, 71). Munir (72) reported that most epidemics in wild small ruminants appear to originate from nearby infected domestic sheep and goats and although there is no plausible evidence of self-sustaining PPRV infection in wild ruminant populations, the potential importance of wildlife in the epidemiology of the disease cannot be ignored. The endemicity of PPR therefore poses a threat, not only to the pastoralists and their livelihoods, but potentially also to the conservation of wildlife and endangered wild small ruminant species (24, 52, 73).

Events/activities that bring together flocks/herds from different farms/localities or introduce sick animals to healthy ones have been identified as major risk factors for PPR in Tanzania and Kenya (35). These activities include communal grazing and housing, the mixing of infected animals with healthy animals in livestock markets, and the introduction of recently purchased or rustled animals to a herd. Similar risk factors for PPR have been identified by other studies in Djibouti (74), Chad (75), India (76), and Pakistan (77, 78). Poor access to veterinary services was identified as a risk factor for PPR in Tanzania (14), and is the bane of livestock production in most of Africa (79). There is a lack of veterinarians or community animal health workers in rural Tanzania, the hub of small ruminant production (29, 80). Consequently, PPR control in rural Tanzania is not highly prioritized (68).

The yearly economic losses attributable to PPR worldwide are enormous (33). Losses due to PPR identified in this review include: mortalities associated with the disease, reduced market value caused by poor body condition, culling, the cost of medication, vaccination, veterinary and labor services, and the cost of embargo on livestock markets imposed by authorities. These agree with those identified in studies from other PPR endemic countries for example in Ethiopia (81, 82), Kenya (83), India (84), and globally (33). The estimated total national loss of income to PPR (92 Million Euros per year) is a huge burden to the Tanzanian economy and underscores the need to eliminate the disease in the country (26).

Control of PPR may be achieved by culling, confinement of infected animals, biosecurity measures to reduce infectious fomites, refusal of imports of sheep and goats from regions suffering outbreaks, and mass vaccination (85). In addition to mass/blanket vaccination, it is also important to target vaccination and sero-surveillance activities at the borders with other PPR endemic areas/countries, to establish immune belts and prevent importation of outbreaks (86). Since the suspected incursion of PPR into Tanzania in 2008, the disease has continued to spread throughout the country, and is now endemic in most regions, despite vaccination campaigns. Mdetele et al. (37) reported a significant increase in antibody detected between pre- and post-vaccination goat and sheep in Northern Tanzania, which suggests that the vaccine may be effective in an outbreak. It is likely therefore, that the inability of vaccination programs to effectively contain the disease may be attributed to other factors such as poor coverage of vaccination programs, lack of control of livestock movement, and the high fecundity due to the dynamic nature of small ruminant populations (26, 87). Herd immunity levels required for successful prevention of PPR transmission is in the range of 70–90% (60), and previous vaccination campaigns in Tanzania may have fallen short of this estimate. Continuous effort is required to maintain high levels of immunity to prevent transmission, especially in small ruminants with a short generation time and high turnover of new/naïve animals (87). Additionally, interference of maternal immunity in young animals, poor vaccine quality, and deficiency in the maintenance of cold chain may also cause vaccination failure (82). Consequently, the reasons for vaccine failure and the persistence of disease transmission in Tanzania should be elucidated. Investigations should be encouraged to further evaluate the barriers to vaccine use, and factors that may affect vaccine efficacy and uptake, including the maintenance of cold-chain storage, and the correct administration. Control by vaccination requires that farmers are aware of the benefits, and that they and their veterinary extension advisors appreciate that frequency of vaccination is related to herd dynamics. Additionally, proper animal identification is necessary for traceability, adequate vaccination coverage, and accurate sero-monitoring (88, 89). Establishing herd status through clinical history and serological testing would be advantageous provided that laboratory access and costs can be managed.

Adequate surveillance of PPR is vital for control and to inform vaccination programs, as demonstrated in countries with successful PPR control policies such as Morocco (90). Indeed, the epidemiological studies accessed for this work covered only few districts/areas of Tanzania, leaving huge areas without data on the status of PPR. For this review, searches were done online only, thus theses, articles and reports not available online were not used for this study. Consequently, the methods used to collect data for this review may have resulted in bias in the study locations, and data from certain locations may have been exempted from this study.

A major hinderance to adequate surveillance is the inability of most antibody tests to distinguish between infected and vaccinated animals (91). This may be overcome with the use of vaccines with DIVA (Differentiating Infected from Vaccinated Animals) capability with their accompanying diagnostic tests, allowing for the discrimination of infected and vaccinated animals (10, 91). This is important for proper planning, execution, and evaluation of control programs (86, 91, 92). Additionally, the use of low-cost, easy to use, point of care diagnostic techniques, and alternative non-invasive sample types may improve surveillance (93–95). At present, there is no official national reference laboratory for PPR in Tanzania, however, the Center for Infectious Diseases and Biologicals (CIDB) of the Tanzania Veterinary Laboratories Agency (TVLA) performs routine testing for PPR and has recently joined a twinning project with OIE Reference Laboratories to improve capacity for PPR diagnosis and expertise (96). International collaborations with organizations such as OIE and FAO should be sought, with local efforts to solve this problem if the target of eradicating PPR globally by 2030 is to be achieved.

This review demonstrates the endemicity of PPR in Tanzania that has major socio-economic impacts on pastoralists and agro-pastoralists in the country, and consequently to the local economy. Uncontrolled animal movement, poor vaccination coverage, mixing of herds/flocks from different farms/localities and sick with healthy animals have aided the transmission and persistence of the disease. Interventions are required to control and eradicate PPR in Tanzania which may be achieved by the collaboration of stakeholders, including: farmers, the Tanzanian government, international organizations (such as FAO and OIE), researchers, and multinational veterinary pharmaceutical companies. An effective widespread/national vaccination campaign must be planned and executed; along with policies aimed at improving awareness of the disease, improving diagnostics, surveillance, disease reporting, and controlling livestock movement; to arrest the spread of the virus and stop the disease incursion into neighboring countries, and achieve the global goal of eradicating PPR by 2030.

## Author Contributions

AE, EM, and RA: conceptualization. AE, RA, EI, and BA: methodology. EI and AO: analysis. EI, BA, RA, AE, AC, AO, EM, and GV: writing and review. AC and GV: funding acquisition. All authors contributed to the article and approved the submitted version.

## Conflict of Interest

The authors declare that this study received funding from Zoetis and the Bill and Melinda Gates Foundation. The funder (Zoetis) had the following involvement with the study: three co-authors (AO, GV, and EM) were Zoetis employees and were involved in study design and the writing of this article.
